# Indications of PCSK9 Inhibitors for Patients at High and Very High Cardiovascular Risk

**DOI:** 10.5935/abc.20180133

**Published:** 2018-07

**Authors:** Paulo Eduardo Ballvé Behr, Emilio Hideyuki Moriguchi, Iran Castro, Luiz Carlos Bodanese, Oscar Pereira Dutra, Paulo Ernesto Leães, Pedro Pimentel Filho

**Affiliations:** 1 Hospital São Lucas da Pontifícia Universidade Católica do Rio Grande do Sul, Porto Alegre, RS - Brazil; 2 Faculdade de Medicina - Universidade Federal do Rio Grande do Sul, Porto Alegre, RS - Brazil; 3 Serviço de Cardiologia - Hospital de Clínicas de Porto Alegre, Porto Alegre - Brazil; 4 Instituto de Cardiologia / Fundação Universitária de Cardiologia, Porto Alegre, RS - Brazil; 5 Irmandade Santa Casa de Misericórdia de Porto Alegre, Porto Alegre, RS – Brazil; 6 Hospital Nossa Senhora da Conceição, Porto Alegre, RS – Brazil

**Keywords:** Cardiovascular Diseases/complications, Cardiovascular Diseases/mortality, Coronary Artery Disease, Proprotein Convertase 9, Hyperlipoproteinemia Type II, Ezetimibe, Simvastatin Drug Combination

Atherosclerotic cardiovascular disease (CVD) is the major cause of ischemic acute coronary events and a significant proportion of ischemic strokes and peripheral artery ischemia. Such events result in significant mortality, physical and/or mental incapacity and costs for the individual and society.^1^

## LDL-C as a risk factor

The causality of plasma LDL-C levels and reduced LDL-C uptake mediated by the LDL-C receptor in the pathophysiology of CVD has been very consistently established.^2^ For patients at very high risk for premature events, such as those with familial hypercholesterolemia (FH), an elevated LDL-C level is an extremely prevalent risk factor.^3^

## Difficulty of achieving the goals with statins

A relevant clinical question is the difficulty of achieving the LDL-C levels recommended by the guidelines for patients at very high cardiovascular (CV) risk. Even using high-potency statins, a substantial proportion of those patients will not achieve the LDL-C target, partially because of the pharmacogenetic effects that determine wide inter-individual variability in the response to statins. This emphasizes the need for an additional reduction in LDL-C levels with new therapeutic options aimed at those atherogenic particles.^4^

## PCSK9 inhibitors

The proprotein convertase subtilisin/kexin type 9 (PCSK9), a member of the serine protease family, plays a central role in the regulation of the liver LDL-C receptor activity. Individuals with mutations in the PCSK9 gene and function loss, and consequent lower LDL-C levels, have a substantially reduced risk of developing coronary artery disease. Inversely, heterozygous individuals for the PCSK9 mutation, with function gain, have a phenotype consistent with FH.

Those findings have stimulated the investigation of the use of PCSK9 inhibitors (PCSK9-I) as an innovative therapeutic alternative to improve the control of elevated LDL-C levels.^5,6^

Several clinical studies with different monoclonal antibodies against circulating PCSK9, both in isolation and combined with statins, have confirmed significant reductions in LDL-C levels, reaching up to 60%.^7,8^

## FOURIER, SPIRE and ODYSSEY

The FOURIER trial, published in 2017, showed a significant reduction in relevant clinical events, such as acute myocardial infarction (AMI) and atherothrombotic ischemic stroke, in patients with established CVD and plainly treated with moderate- and high-potency statins, associated or not with ezetimibe. The median LDL-C level was 92 mg/dL, and patients receiving evolocumab reached a median LDL-C level of 30 mg/dL. Safety data showed no significant adverse effect, except for injection-site reactions, in the median follow-up of 2.2 years.^9^

The recently published EBBINGHAUS trial, assessing patients who received either PCSK9-I or placebo in addition to statin therapy, has shown no significant difference in cognitive function, even in individuals with very-low LDL-C levels.^10^

Another study using the monoclonal antibody bococizumab has shown a significant reduction in CV events, which is aligned with the result obtained with evolocumab in the FOURIER trial. However, the SPIRE trial was interrupted because of the development of high rates of antidrug antibodies and consequent reduction in the therapeutic response.^11^

The ODYSSEY trial, recently presented as a late-breaking clinical trial at the American College of Cardiology Scientific Session, has assessed patients who had acute coronary syndrome within 1 to 12 months before randomization. All individuals were on moderate- and high-potency statins, associated or not with ezetimibe. The mean follow-up was 2.8 years.^12^ The trial has shown a significant reduction in non-fatal AMI, unstable angina and ischemic stroke in patients randomized to receive the PCSK9-I alirocumab. The subgroup of individuals with LDL-C levels greater than or equal to 100 mg/dL (already treated with statins) and receiving alirocumab had the highest benefit, with a 29% reduction in total mortality as compared to placebo.

## Cost *versus* benefit of new therapies

Although the advent of precision medicine and the innovative treatments have guided to an individualized approach in prevention and patient’s management, the financial restrictions to the progressive increase in health system costs worldwide often requires balancing the therapeutic benefit and the cost of a certain intervention.

## Brazilian guideline

The recently updated Brazilian Guideline on Dyslipidemias and Atherosclerosis Prevention recommends the use of PCSK9-I (evolocumab and alirocumab) only to patients at high CV risk, receiving optimized treatment with statins at the highest tolerated dose, either associated or not with ezetimibe, and who have not met the recommended LDL-C or non-HDL-C targets.^13^

The Brazilian guideline, however, does not indicate which individuals will benefit most from the use of that new class of drugs.

Some studies have demonstrated that the quantification of the absolute benefit of an additional therapy is an important factor to support the clinical decision of using or not the new treatment. In addition, financial aspects should be taken into account, but so far cost-effectiveness analyses of the PCSK9-I in Brazil have not been made.^14^

Considering that the costs of PCSK9-I are greater than those of the other drugs for the treatment of CVD, it is important to identify among high-risk individuals those whose treatment is associated with greater clinical relevance, which can be estimated by the number needed to treat (NNT) to prevent the first outcome within a certain time.^15^

In addition, calculating the NNT can help identify groups of patients who will benefit most from the addition of a non-statin therapy, by combining absolute risk and LDL-C thresholds.

In this position statement of the Atherosclerosis Department of the Rio Grande do Sul Society of Cardiology, the selection of patients to use PCSK9-I is more conservative than that of most current guidelines on the use of those drugs. Thus, it is worth emphasizing that the use of antibodies against PCSK9 for individuals who do not meet the criteria presented in this document is not contraindicated, because the therapeutic decision involves clinical judgement and consensus between physicians and patients.

Therefore, this position statement was aimed at identifying the individuals who will benefit most from the use of that new class of drugs to treat hypercholesterolemia.

This first position statement will not address indications of that new class of drugs for statin-intolerant individuals or those on high-risk primary prevention, such as FH.

Considering that evolocumab and alirocumab consistently reduce LDL-C levels by 50% at least, two factors should be observed to quantify the benefit of the treatment: the individuals’ clinical characteristics and the LDL-C levels obtained after maximum treatment with statin/ezetimibe.

### 1) Clinical characteristics of the patients

The clinical characteristics of the patients at CV risk should be identified based on the absolute risk of CV events in 10 years.^16^ The greatest benefit derived from the use of PCSK9-I is obtained in individuals at CV risk higher than 20% in 10 years. Thus, patients with previous coronary events or procedures, stroke or aortic aneurysm are classified as “high risk” (20-29% in 10 years).

Patients at “very high risk” for CV events (over 30% in 10 years) are those with recurrent acute coronary syndrome, repeated arterial revascularization or repeated strokes within the first year from the initial event. Advanced age, and the association of diabetes or peripheral occlusive arterial disease are aggravating factors.

### 2) LDL-C cutoff points after maximum treatment with statin/ezetimibe

In addition to the patients’ clinical characteristics, the LDL-C cutoff points from which the treatment with PCSK9-I provides the greatest benefits should be indicated.

The FOURIER trial has shown that even individuals in the lowest quartile of LDL-C levels had a significant reduction in CV events when receiving evolocumab.

**Figure 1:**
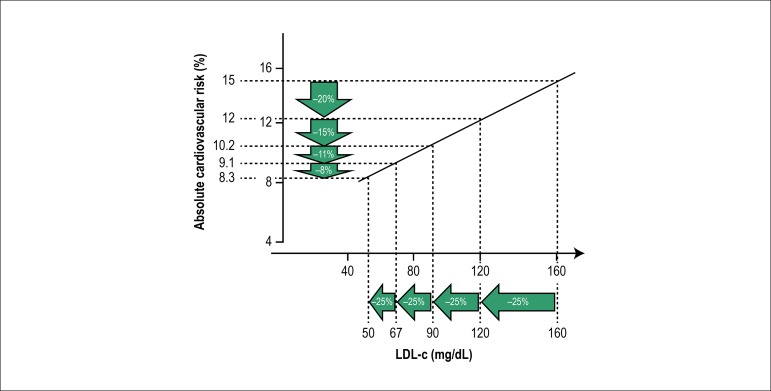
Absolute risk reduction for the same relative LDL-C level reduction from different initial LDL-C levels. (Reprint with permission from Oxford University Press).^17^

However, when assessing that variable, it is worth noting that the reduction in the absolute risk for the same relative reduction in LDL-C level will be smaller when the baseline LDL-C level is lower (Figure 1). In other words, the higher the LDL-C level after treatment with statins/ezetimibe, the greater the benefit deriving from the treatment with PCSK9-I and the smaller the NNT.^17^

The relative reduction in CV events resulting from the use of statins, ezetimibe and monoclonal antibodies against PCSK9 has shown consistency with the relationship reported in the Cholesterol Treatment Trialists meta-analysis, in which every 39-mg/dL reduction in LDL-C was associated with a 21% reduction in major CV events.^18^

## Criteria to support decision-making

By associating the two variables presented in an excellent analysis and according to the CV risk and LDL-C levels of patients receiving treatment with statins, Robinson et al.^16^ have estimated the NNTs in 5 years to prevent a CV event.^16^ (Table 1) 

**Table 1:** NNT in 5 years to prevent a cardiovascular (CV) event in "high" and "very high CV risk" individuals receiving treatment with high-potency statins by adding the PCSK9 inhibitor (PCSK9-I)

Initial LDL-C	50% reduction in LDL-C (with PCSK9-I)	65% reduction in LDL-C (with PCSK9-I)
**High risk (20-29% risk of ACVD in 10 years)**		
190	19	15
160	23	18
130	28	22
100	37	28
70	53	40
**Very high risk (risk of ACVD in 10 years ≥ 30%)**		
190	13	10
160	15	12
130	19	15
100	25	19
70	35	27

LDL-C: low-density-lipoprotein cholesterol; PCSK9: proprotein convertase subtilisin/kexin type 9; ACVD: atherosclerotic cardiovascular disease. (Table adapted with permission from Elsevier).^16^

Although there is consensus that NNTs up to 50 are acceptable^19^ for new interventions, it is worth noting that PCSK9-I are high-cost drugs. On the other hand, NNTs under 20 are rarely obtained for interventions to treat or prevent CVD resulting from the current studies.^20^

Assuming that PCSK9-I consistently reduce LDL-C levels by at least 50%, we consider that NNTs under 30 are totally acceptable and identify a subgroup of individuals who will greatly benefit from receiving that new class of drugs.

Therefore, patients at high CV risk, plainly treated with high-potency statin associated with ezetimibe, and whose LDL-C levels are higher than 130 mg/dL, will significantly reduce their risk of CV events by adding PCSK9-I to their treatment.

Similarly, individuals at very high CV risk, treated with statin and ezetimibe, and whose LDL-C levels are higher than 100 mg/dl, have a very good chance of significantly reducing outcomes and the residual CV risk by adding that new class of drugs to their treatment.

## Conclusion

This position statement of the Atherosclerosis Department of the Rio Grande do Sul Society of Cardiology identifies patients who can derive the greatest secondary clinical benefit from PCSK9 inhibition. Those patients have higher CV risk associated with the highest probability of achieving a significant LDL-C reduction. In addition, this document takes into account the financial limitations of the healthcare system and the current economic scenario.

It is worth emphasizing that the use of antibodies against PCSK9 for individuals who do not meet the criteria presented in this document is not contraindicated, because the therapeutic decision involves clinical judgement and consensus between physicians and patients.
